# Characterization of gap-plasmon based metasurfaces using scanning differential heterodyne microscopy

**DOI:** 10.1038/s41598-020-70395-2

**Published:** 2020-08-11

**Authors:** Ildar M. Akhmedzhanov, Rucha A. Deshpande, Dmitry V. Baranov, Sergey I. Bozhevolnyi

**Affiliations:** 1 Prokhorov General Physics Institute of the Russian Academy of Sciences, Vavilov str. 38, 119991 Moscow, Russia; 2 Centre for Nano Optics, University of Southern Denmark, Campusvej 55, 5230 Odense M, Denmark

**Keywords:** Metamaterials, Scanning probe microscopy

## Abstract

Optical phase-gradient metasurfaces, whose unique capabilities are based on the possibility to arbitrarily control the phase of reflected/transmitted light at the subwavelength scale, are seldom characterized with direct measurements of phase gradients. Using numerical simulations and experimental measurements, we exploit the technique of scanning differential heterodyne microscopy (SDHM) for direct phase and amplitude characterization of gap-plasmon based optical metasurfaces. Two metasurface configurations utilizing the third-order gap surface plasmon (GSP) resonance, representing a binary grating and linear phase gradient, are experimentally characterized with the SDHM operating at the light wavelength of 633 nm. Comparing the experimental performances of these GSP metasurfaces with those expected from the phase and amplitude profiles reconstructed from the SDHM measurements, we verify the efficiency and accuracy of the developed SDHM characterization approach for direct inspection of GSP reflective metasurfaces.

## Introduction

Gap-plasmon based phase-gradient metasurfaces operating in reflection allow one to selectively engineer properties of unit cells and integrate multiple functionalities in a single device with excellent performance, realizing thereby diverse flat multifunctional optical components^1^, ranging from focusing polarization beam splitters^2^ and vector-beam generators^3^ to spectropolarimeters^4^ and unidirectional couplers for surface waves^5^. Achieving excellent performance faces however serious challenges in both ingenious design, which is based on accurate modelling of complicated electromagnetic scattering problems, and laborious nanofabrication, which is plagued by technological imperfections that could be related to deviations of geometrical parameters of fabricated nanostructures from the designed ones or material properties, such as the metal and/or dielectric susceptibilities, from the handbook data^6^. Considering the latter, degradation in the overall performance of fabricated components is often related to the technological imperfections, although it is very difficult to pinpoint the critical issue and identify the origin of deviations in the observed performance with respect to that expected from the design simulations. The reconstruction of amplitude and phase distributions of fields reflected by fabricated metasurfaces so as to allow one to compare these characteristics with the designed ones (instead of comparing their performances) requires the development of appropriate characterization techniques. In fact, this development is crucial for further progress towards the implementation of practical flat optical components that would have to compete in quality with conventional (very well developed and characterized) optical components.

Several characterization approaches suitable for controlling the performance of optical metasurfaces were recently proposed and experimentally tested^7–9^, with all methods utilizing different physical principles and revealing different limitations in their performances. The most recently suggested technique, which involves scattering near-field optical microscopy with phase-resolved detection allowing for characterization of individual surface elements^9^, has limitations associated with the necessity of back-side illumination of metasurfaces that would normally be illuminated from the front side. This approach requires thereby the development of a special recalculation procedure when treating the experimentally obtained phase and amplitude distributions^9^. In this work, we conduct experimental investigations of an alternative approach to the characterization of reflective metasurfaces that is based on the usage of scanning differential heterodyne microscopy (SDHM). The SDHM application to the characterization of reflective metasurfaces was recently proposed and considered using theoretical considerations and numerical simulations^10^. It is however clear that there are many issues, such as influence of noise, limited resolution and experimental inaccuracies, whose importance for accurate SDHM characterization of metasurfaces can only be evaluated when the SDHM technique is applied in practice.

Here we perform the SDHM characterization at the light wavelength of 633 nm of two different metasurface configurations utilizing the third-order gap surface plasmon (GSP) resonance^11^, representing a binary grating and linear phase gradient. The basic element in these GSP metasurfaces represents a 450 × 450 nm^2^ unit cell consisting of an optically thick gold substrate covered with a 40-nm-thin silica spacer layer loaded with a 50-nm-high gold nanobrick having variable lateral dimensions. The third order GSP resonance provides the possibility of tuning the phase and amplitude of the reflected light by adjusting (relatively large) lateral nanobrick dimensions^11^. Depending on the desired metasurface functionality, individual cells with, in general, differently sized nanobricks are combined in a supercell, thus forming the structured GSP metasurface. The binary grating configuration consists of the periodically arranged stripes containing ten identical individual cells separated by unpatterned substrate areas, whereas the linear phase-gradient metasurface comprises periodic supercells containing five differently sized phase-shifting individual cells, each repeated three times in order to decrease the beam steering angle^12^. Ideally, the phase shift should be the same for each three identical neighbour cells. However, due to the near-field coupling between individual cells^9^ that is very difficult to incorporate in the design simulations^12^ and inevitable technological fabrication-induced imperfections, the real phase shifts deviate from the designed values leading to deviations of the diffraction characteristics from the targeted ones. For the purpose of identifying specific imperfection areas in the fabricated reflective metasurfaces we suggest using the SDHM as a reliable and robust tool.

Our main aim is to experimentally characterize the SDHM responses for two specifically designed GSP metasurfaces and conduct their corresponding interpretation in order to determine malfunctioning of individual elements by comparing the measured and calculated (using the amplitude and phase reconstruction) far-field diffraction characteristics.

## SDHM response basics and interpretation

### Experimental setup and microscope response

**Figure 1:**
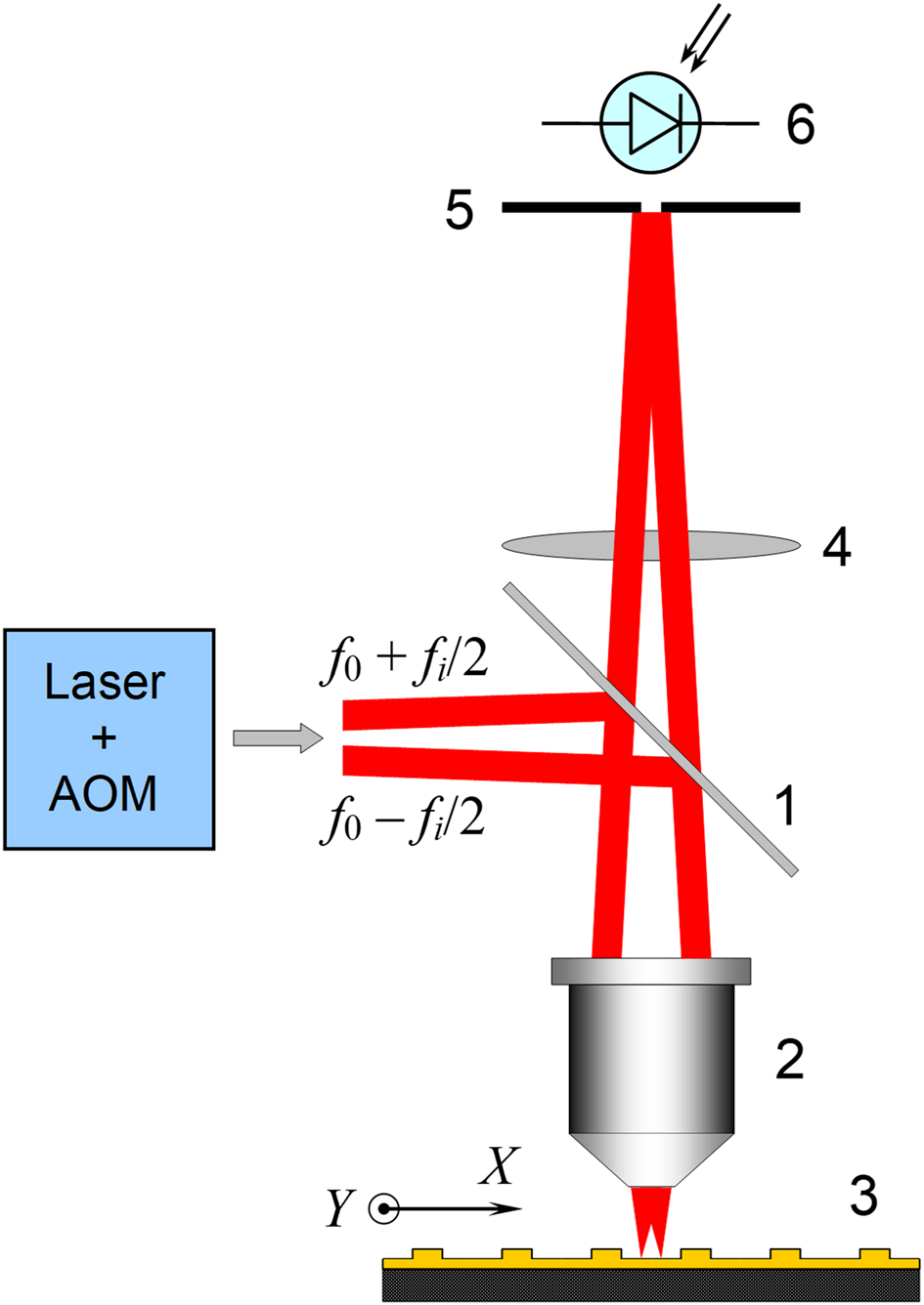
Optical scheme of a scanning differential heterodyne microscope: (1) beam splitter; (2) microobjective 40^×^/0.65; (3) substrate with an object; (4) positive lens; (5) pinhole in the Fourier plane; (6) photodetector. Probing beams at different frequencies come from acousto-optic modulator (AOM) driven by two-frequency signal.

**Figure 2:**
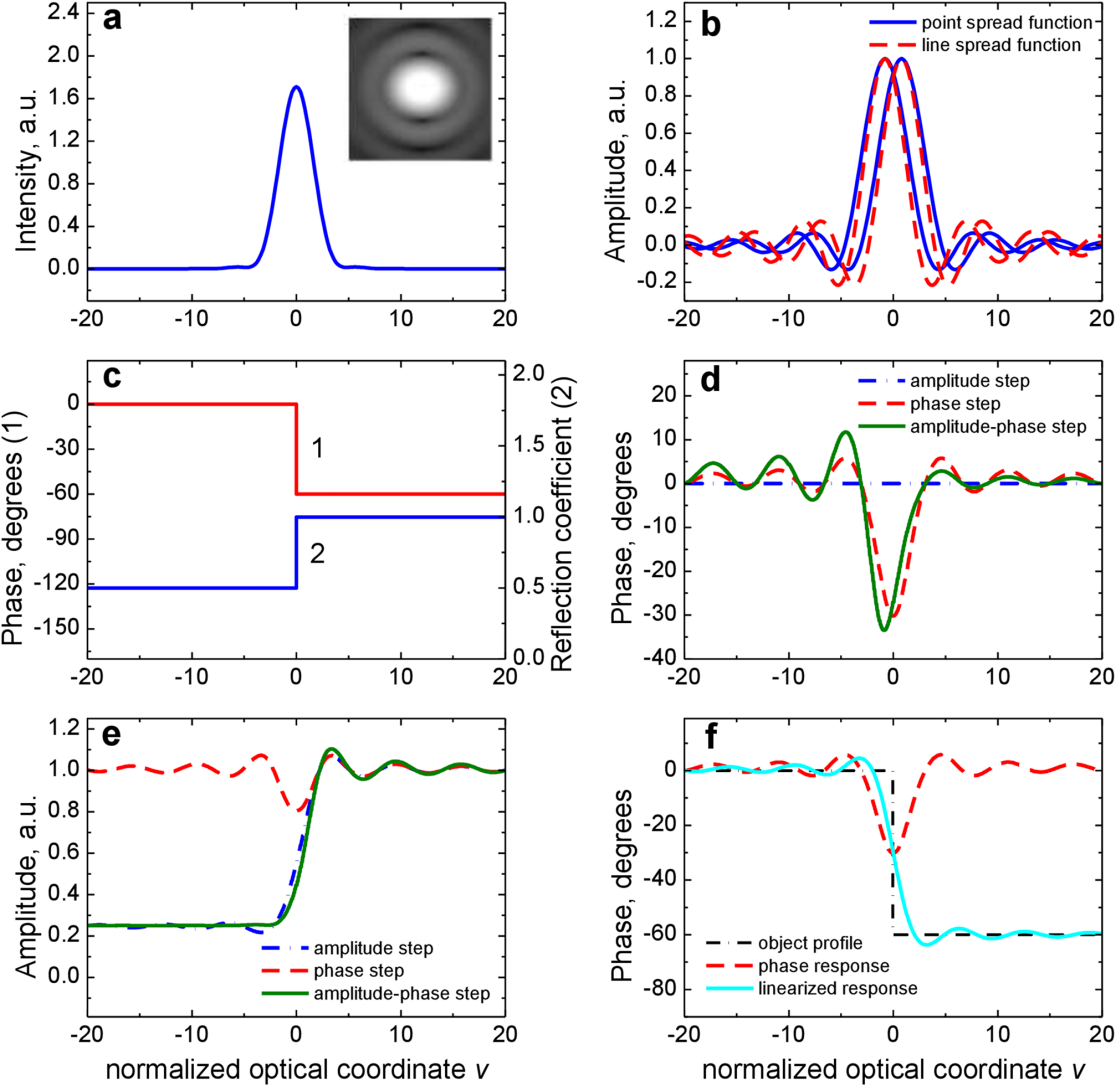
Probing beams, point and line spread function of the SDHM and the microscope responses to step objects. (**a**) Intensity profile of the probing beams and 2D grey-scale intensity (inset). (**b**) Amplitude profiles of the separated probing beams as the point spread function (solid line) and the line spread function (dashed line). (**c**) Model profiles of phase object (1) and amplitude object (2). (**d**) SDHM phase response to amplitude-phase step (solid line), phase step (dashed line) and amplitude step (dash-dotted line). (**e**) SDHM amplitude response to amplitude-phase step (solid line), phase step (dashed line) and amplitude step (dash-dotted line). (**f**) SDHM phase response to phase object (dashed line) and appropriate phase image retrieval based on linearization algorithm (solid line).

The measurements were carried out on SDHM experimental setup (Fig. 1) based on a well-known common path scheme of Mach–Zehnder interferometer with a He–Ne laser at wavelength *λ* = 633 nm as a light source^13^. Two diffraction probe beams are formed by a Bragg acousto-optic modulator with a centre frequency *f*_0_ = 160 MHz. The probe beams are shifted by frequencies *f*_0_ − *f*_*i*_/2 and *f*_0_ + *f*_*i*_/2 and propagate at a small angle relative to each other. Thus the difference (or heterodyne) frequency is *f*_*i*_, tunable in the range 0.1–1 MHz The microobjective (2) with numerical aperture NA = 0.65 forms two partially overlapping spots spaced by the interval *δ* in *XY* plane dependent on the frequency *f*_*i*_. The simulated intensity profile of the focused spots for the actual value of *δ* is shown in Fig. 2a. The optical field amplitude distribution of each probing beam is characterized by a FWHM size 2*w* ≈ 0.7 μm (Fig. 2b). The light intensity reflected from an object is recorded by the point photodetector (6) in the Fourier plane of the microobjective after passing the beam splitter (1). Since the original Fourier plane is located in the vicinity of the microobjective, the lens (4) images it to the registration plane with the pinhole (5). The object is scanned along the line passing through the centres of the probe beams coinciding with *X* axis. The scanning step is equal to the distance between focused beams *δ*. The phase and amplitude of photodetector signal at heterodyne frequency are modulated by object properties. The phase is registered by a phasemeter with a signal of reference photodetector (not shown in Fig. 1) at the same frequency and the amplitude is measured by a selective voltmeter. The dependences of the amplitude and phase of the photodetector current at the heterodyne frequency *f*_*i*_ on the object coordinate *x*_*s*_ we call the amplitude and phase response of the SDHM to an object under investigation. The complex response *D*(*x*_*s*_) of SDHM in the exponential representation is expressed as

1 $$i(x_{s} ,t) = i_{0} {\text{Re}} \left\{ {D(x_{s} )\exp (2\pi jf_{i} t)} \right\},$$

where *i*_0_ is the photodetector current for a plane substrate, *f*_*i*_ is the heterodyne frequency and

2 $$D(x_{s} ) = A(x_{s} )\exp \left[ {i\phi (x_{s} )} \right]$$

Here the phase response *φ*(*x*_*s*_) = arg *D*(*x*_*s*_) and the amplitude response *A*(*x*_*s*_) =|*D*(*x*_*s*_)|. The complex response is normalized in such a way that *D*(*x*_*s*_) = 1 for the unpatterned area.

### Response modelling for step-like object

Most objects are represented as a combination of amplitude-phase steps. This also can be applied to metasurfaces. Therefore, to get clear understanding of the nature of the experimental responses of the SDHM first let us consider calculated responses for amplitude-phase steps with a difference in the phase reflection coefficient Δ*φ* = 60° and amplitude reflection coefficient Δ*A* = 0.5 (Fig. 2c). The responses were obtained within the thin amplitude-phase screen approximation^14^ and the line spread function (LSF) approach. Using LSF instead of the point spread function (PSF) means that hereinafter we neglect the dependence of the optical properties of the object on the *Y* axis. This is justified since for the experimental samples used, the linear scale along the *Y* axis is determined by the size of the elementary plasmon cell (450 nm) which is significantly less than the characteristic size of focused probing laser beams (~ 1 μm). As for the PSF, it is determined by the shape of the focused probe beams on the surface of the object. For the uniform illumination of the entrance aperture of the microobjective used in the experiment, the amplitude distribution of the optical field for each probe beam at the focus is determined by the first order Bessel function of the first kind which leads in turn to the sinc function for the LSF^15^ (see Fig. 2b). As usual in such calculations the linear dimensions are expressed in normalized optical coordinate *v* = 2*πx*NA/*λ*, where *x* is the linear coordinate^15^. For modelling the typical SDHM focused spot parameters were used: *δ* = *w* = 0.5 μm. The presented dependences allow us to make the following main conclusions about the properties of the SDHM response to objects of this type:

1. The phase response to the step has a pulsed form with a characteristic distribution width of the order of the LSF width (Fig. 2d).

2. The magnitude of the phase response is given by^10^:

3 $$\phi_{\max } = \frac{\delta }{2w}|\Delta \phi |,$$

where 2*w* is the full width of the focused probe beam, *δ* is the interval between two focused beams. In our case, if Δ*φ* = 60° and *δ* = *w* = 0.5 μm we obtain *φ*_max_ = 30° that is in good agreement with the result of the exact calculation shown in Fig. 2d for both phase and amplitude-phase steps.

3. Amplitude response, except for the case of phase only object, reproduces the shape of a step with characteristic blurring of the LSF width (Fig. 2e).

4. The magnitude of the amplitude response can be estimated by squaring the amplitude reflection coefficient of the original step object.

On this basis as it will be shown below, it is possible to make a rapid interpretation of the SDHM response and estimate the parameters of a step amplitude-phase object. For more accurate recovery of the amplitude-phase reflection coefficient, as a function of the coordinate, and for more complicated optical profiles generally one should solve the inverse problem for the SDHM response. Different approaches exist for this purpose depending on the type of an object under investigation^16^. In our opinion, the method based on the linearization of the SDHM response, which is obviously nonlinear with respect to reflection coefficient^10^, is of particular interest. The nonlinear nature of the SDHM response leads, in general case, to a complicated dependence of the amplitude-phase response on the actual amplitude-phase properties of the reflecting surface. However, based on the fact that at least one case exists where the amplitude and phase properties of the surface separately affect the amplitude response and phase one^10^, we decided to apply following version of the linearization algorithm:

1. The phase profile of the reflection coefficient is obtained by integrating, that is, summing, the discrete samples of the phase response recorded at object scanning.
2. The amplitude profile of the reflection coefficient is obtained by calculating a square root of the amplitude response.

As an example, the model phase retrieval for the phase step object is represented in Fig. 2f. For real experimental objects the correctness of this method should be checked up and this is one of the aims of our investigation. If the reflection coefficient is correctly recovered, then its squared Fourier transform should give the diffraction pattern in the far-field zone, which allows easy experimental verification.

## Results

**Figure 3:**
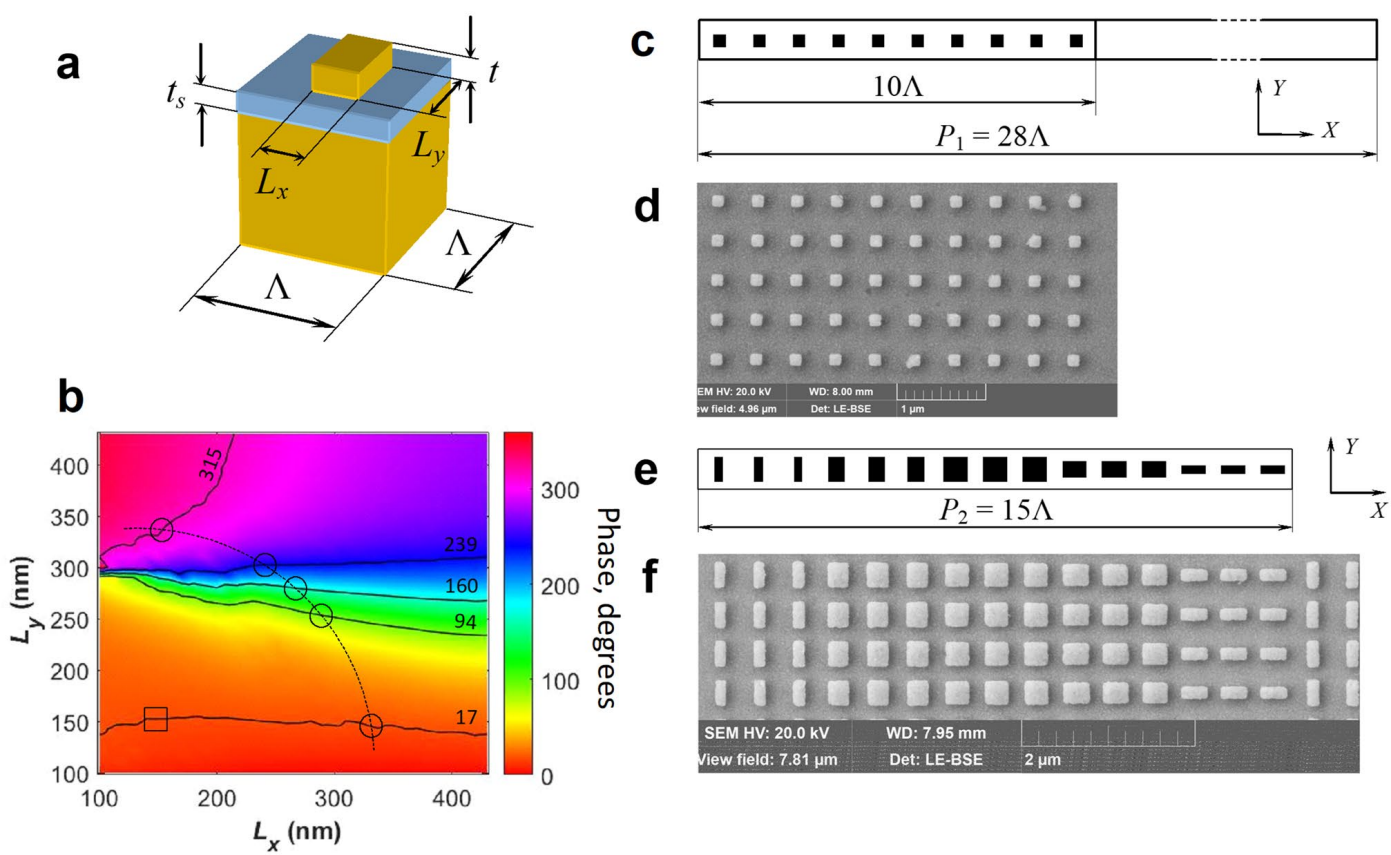
Designed and fabricated metasurface objects. (**a**) Sketch of basic unit cell consisting of a gold nanobrick of *L*_*x*_ × *L*_*y*_ size with high *t* = 50 nm on the top of a glass spacer with thickness *t*_*s*_ = 40 nm and thick golden layer below in period Λ = 450 nm. (**b**) Calculated phase map for nanobricks of varying widths at wavelength *λ* = 633 nm for normally incident TE polarized light. The designed phase values (17°, 94°, 160°, 239°, 315°) are shown in contour lines. The element widths are indicated by the square for binary metasurface grating and by the circles for gradient metasurface The elements of gradient metasurface cover the phase up to 2*π* in five steps of ~ 72°, 1.2 rad. The *L*_*x*_ and *L*_*y*_ widths of chosen elements shown in circles are 153 × 333, 253 × 300, 273 × 280, 300 × 253 and 320 × 146 nm^2^. The value for element shown in the square is 150 × 150 nm^2^. (**c**) Top-view of one period of designed binary grating. (**d**) Representative SEM image of 10 unit cells within one period of fabricated binary grating. (**e**) Top-view of designed super cell of phase-gradient metasurface consisting of 5 sets of 3 unit cells. (**f**) Representative SEM image of super cells in the fabricated phase-gradient metasurface.

The schematic of the studied GSP resonance configurations are presented in Fig. 3. The general information about composition of used metasurfaces was already provided in the Introduction section. According to numerical calculations^12^ the phase difference between incident and reflected light beam can be tuned up to 2*π* by varying lateral dimensions of nanobricks. Two objects were designed for testing using SDHM by considering the set of individual nanobrick elements (unit cells) that produce phase gradient and form the grating. For the considered metasurface configuration data (Fig. 3a), we calculated the amplitude and phase of the reflected light (under normal incidence) with COMSOL Multiphysics software based on finite-element method, which is a rather standard procedure (see, for example^11,12^). We then chose the nanobrick dimensions to ensure the designed phase difference as indicated in Fig. 3b by a square (the designed phase difference of 17° or 0.29 rad) and circles (the designed phase differences of 72° or 1.2 rad). Here, we describe the experimental objects in detail. The first object is the binary grating composed of unit cells with the length Λ = 450 nm ensuring the designed phase difference in reflected light of 17° or 0.29 rad for *λ* = 633 nm. The schematic and fabricated binary grating are shown in Fig. 3c,d, respectively. The period of the binary grating is about 12.6 μm and consists of 10 unit cells containing identical nanobricks and free from nanobricks substrate space of 18Λ in width. Thus, we have designed the amplitude-phase binary grating with the stripe spacing *P*_1_ = 12.6 μm inserting reflection phase difference of 17^°^ or 0.29 rad and reflection amplitude difference ~ 0.9 within the grating period. The second object was designed as a phase-gradient metasurface^17^, which is schematically shown in Fig. 3e. Fabricated phase-gradient metasurface is shown in Fig. 3f. According to design characteristics, the phase-gradient metasurface can be represented as a reflecting surface in which the phase reflection coefficient varies periodically with the period value *P*_2_ featuring 5 discretization levels that cover the 2*π* phase range. The phase steps are designed to be ~ 1.2 rad for for the *Y* polarized incident light. The reflection amplitude coefficient is within the range 0.5 ÷ 0.9 and different for each level. One period of metasurface represents one supercell consisting of 5 sets, and each set is composed of 3 unit cells. Thus, the grating period is equal to the supercell length: *P*_2_ = 15Λ = 6.75 μm with the designed total phase increment of 2*π*. Both test objects are well suited for testing SDHM as a tool for characterization of metasurfaces which should be able to reveal the difference between designed and fabricated metasurface samples.

Two methods were used to interpret the responses of the SDHM and determine the amplitude-phase profile of objects. The first method consists in estimating the phase profile of the object according to Eq. (3) and modeling the original object in accordance with this estimation. In the second method the amplitude-phase linear response is calculated from the experimental differential complex response of the microscope. The obtained linear response is supposed to represent the amplitude-phase profile of the object within the thin phase screen approximation. The results obtained with both methods will be compared with each other.

**Figure 4:**
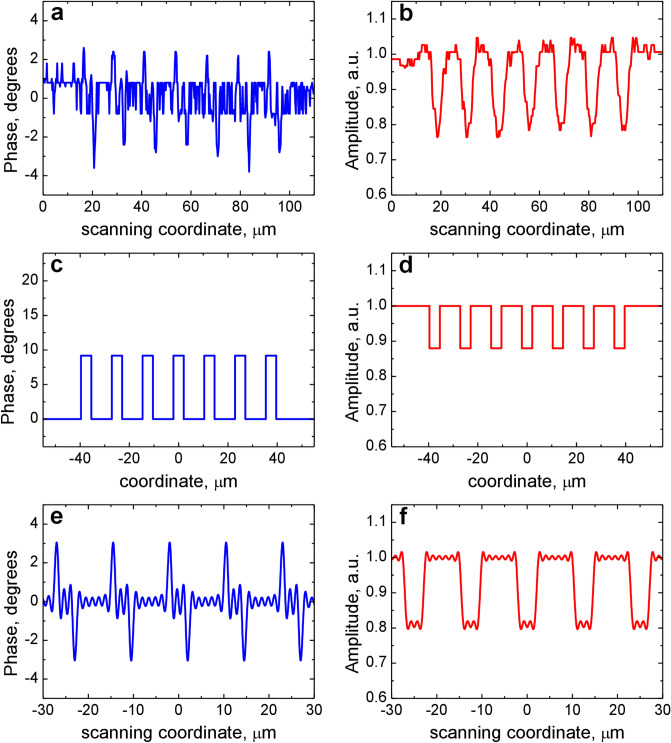
Direct profile reconstruction for the binary metasurface grating. (**a**) Experimental SDHM phase response. (**b**) Experimental SDHM amplitude response. (**c**) Binary phase object profile calculated from the SDHM response with Eq. (3). (**d**) Binary amplitude object profile derived from the SDHM amplitude response by calculating a square root. (**e**) SDHM phase response (compare with **a**) calculated from the profile in (**c**, **d**). (**f**) SDHM amplitude response (compare with **b**) calculated from the profile in (**c**, **d**).

First, we describe the results obtained for the binary grating as a simpler structure. The grating structure with a length of 80 μm along the *X* axis has 7 binary stripes characterized by the phase magnitude Δ*φ* and the amplitude magnitude Δ*A* to be determined. The grating is homogeneous along the *Y* axis and its *y*-length is also equal to 80 μm. The experimental phase response to the grating (Fig. 4a) has 7 positive pulses and 7 negative ones (2 pulses per a stripe) with the magnitude *φ*_max_ = (3.0 ± 0.5)°. Since the positive and negative pulses are separated laterally, the phase response consists of individual responses to 14 steps and each step is resolved by the microscope. Therefore, in this case, we can use the estimation of Eq. (3) derived for a step-like object. The amplitude response (Fig. 4b) has 7 pulses with the magnitude Δ*A*_*d*_ = 0.77 ± 0.01 (Fig. 4b). Based on these data the estimate of the phase magnitude of the grating stripe was made with Eq. (3): Δ*φ* = (2*w*/*δ*)*φ*_max_ = 9.5°. This value differs considerably from the design phase step of 17^0^, a difference that can be due to a number of reasons, including deviations of geometrical parameters such as nanobrick sizes and the spacer thickness. The amplitude magnitude was calculated as Δ*A* = (Δ*A*_*d*_)^1/2^ = 0.88. These values were obtained for the diameter of the probe spot 2*w* = 0.7 μm and the interval between the probe spots *δ* = 0.22 μm. Using the values Δ*φ* and Δ*A*, the amplitude-phase profile of the binary grating was simulated (Fig. 4c,d), from which the complex response of the SDHM was calculated using the scalar theory in the approximation of a thin phase screen. The calculated responses are shown in Fig. 4e,f. When comparing the experimental and model responses, good agreement is observed in the magnitudes of the phase and amplitude responses. It should be noted that the source of the observed noise in the experimental phase and amplitude responses is due to the analog-to-digital converter included in the signal processing system. In our experiments, the phase noise was ≈1.5°, so that the phase fluctuated within the interval of [− 0.8°, 0.8°], and did not depend on the magnitude of a phase response, i.e., the signal-to-noise ratio can become rather low when measuring weak phase objects.

**Figure 5:**
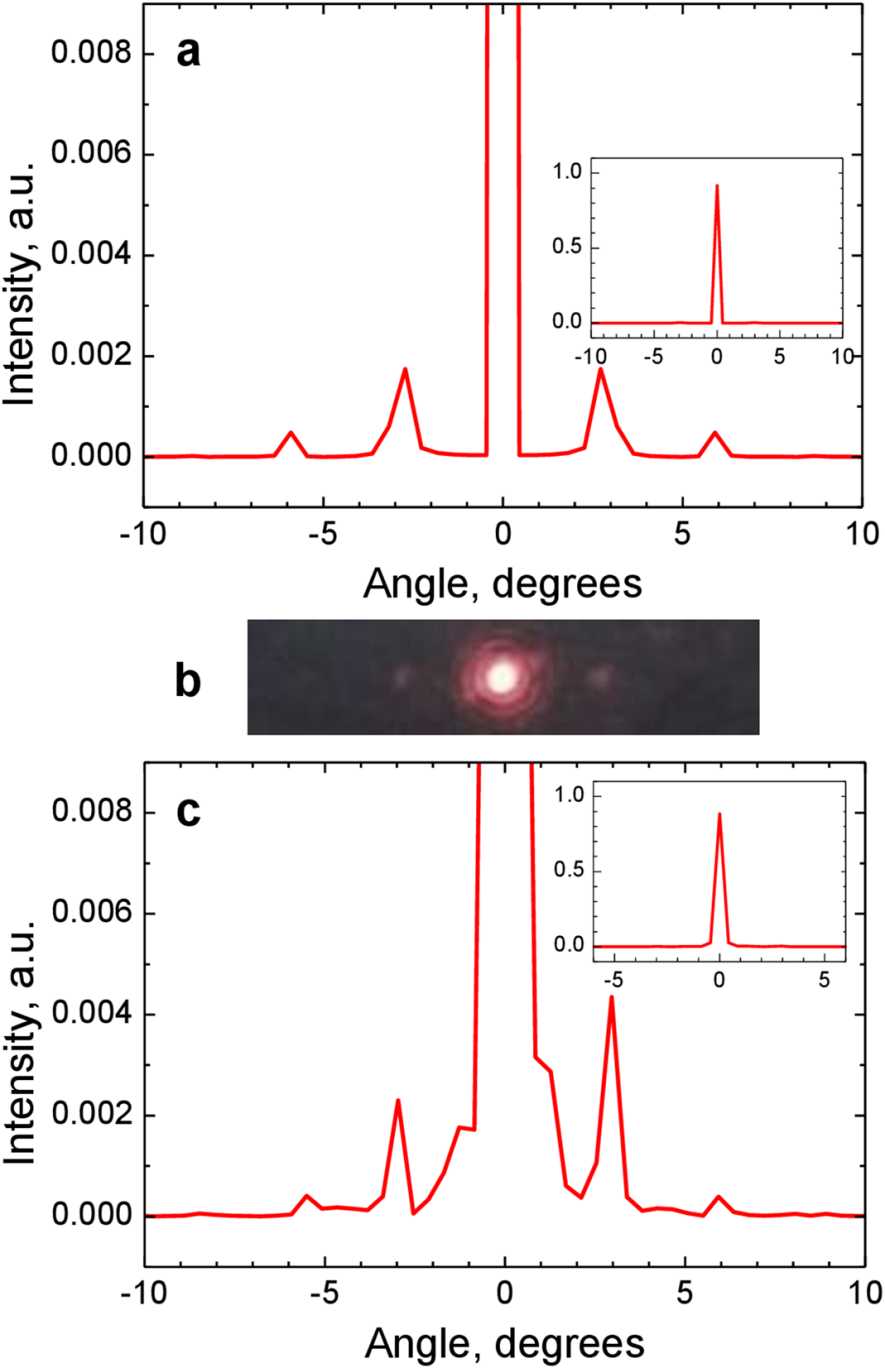
Simulated spectrum intensity and experimental diffraction pattern. (a) Intensity profile for the modelling binary grating profile. (**b**) Experimentally registered diffraction pattern of the binary grating. (**c**) Intensity profile for the experimentally reconstructed grating profile. The full-scale intensity is shown in the inset of each graph.

The model profile of the binary grating (Fig. 4c,d) retrieved from the experimental results (Fig. 4a,b) was used to calculate the efficiencies of diffraction orders for an infinitely extended grating being illuminated by a plane wave with a wavelength of *λ* = 633 nm. The far-field diffraction intensity profile was obtained as the squared absolute value of the Fourier transform of the amplitude-phase profile (Fig. 5a). The first diffraction orders have the same efficiency of 0.2%, the second orders are of 3 times less. To verify this estimation, the experimental measurements of the diffraction efficiencies of the binary grating were carried out. The main condition for the correct measurement was matching the size of the illuminating beam to the grating size (80 × 80 *μ*m^2^). The illumination scheme consisting of a lens, a microobjective 40^×^/0.65 and an iris diaphragm forms a 70-*μ*m-diameter laser beam. The efficiency of diffraction orders was determined by the following relation: *R*_*n*_ = (*I*_*n*_/*I*_0_)100%, where *I*_0_ is the intensity of the beam incident on the grating and *I*_*n*_ is the intensity of the *n*th diffraction order. The optical image of the far-field diffraction pattern is shown in Fig. 5b, and the experimental diffraction efficiencies are *R*_−1_ = 0.3%, *R*_+1_ = 0.4%. Second orders were negligible and could not be measured because of insufficient sensitivity of the equipment. These measured diffraction efficiencies, although not equal, are close to those (0.2%) expected from the phase profile estimated on the basis of the experimentally measured SDHM phase response as described above. At any rate, the designed phase profile with the phase step of 17° would have resulted in significantly stronger diffraction orders.

**Figure 6:**
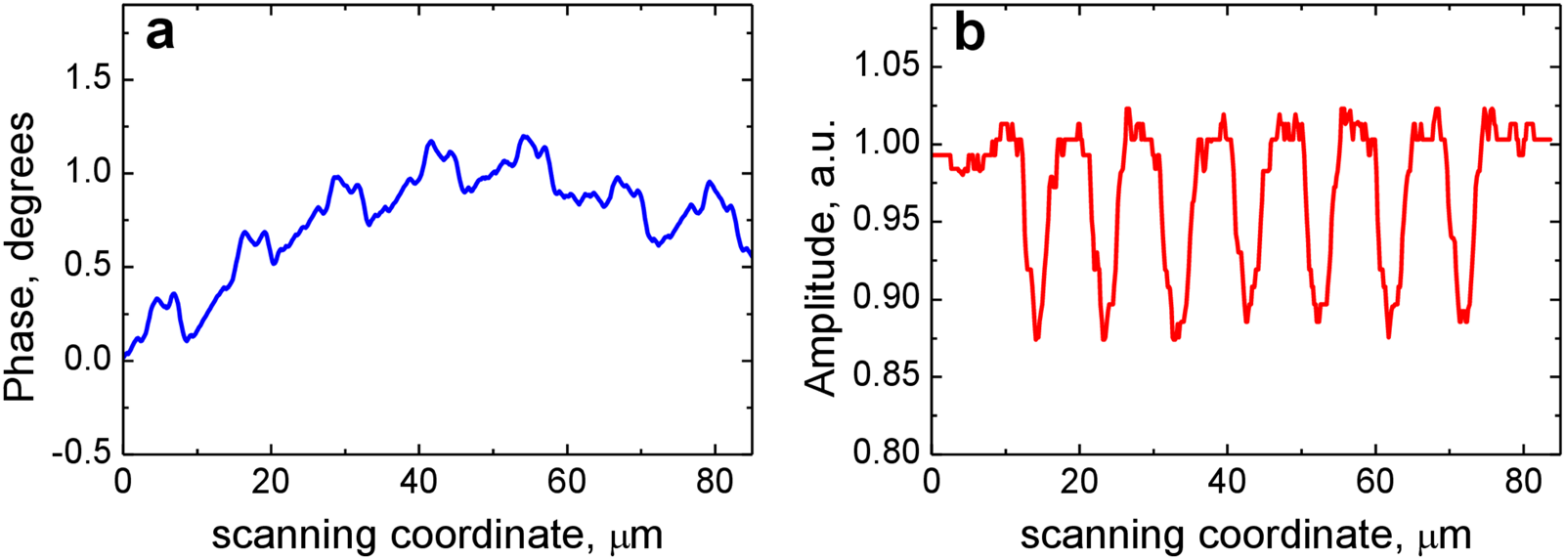
Amplitude-phase profile of the binary grating. Linearized SDHM phase (**a**) and amplitude (**b**) response are calculated from the experimental response shown in Fig. 4a,b.

In this regard, the next step in processing the complex SDHM response on the binary grating was the linearization of the response in order to determine the amplitude-phase profile of the grating more accurately. The differential response shown in Fig. 4a,b was processed using the linearization procedure described in Sect. 2. The obtained linear response is shown in Fig. 6a,b. As mentioned above, we expect that this response should result in a more adequate amplitude-phase profile of the binary grating than the binary profile in Fig. 4c,d, since the linearized response is evaluated directly from the experimental SDHM phase response. The Fourier spectrum of the amplitude-phase profile obtained as a result of linearization procedure does in fact produce more adequate efficiency values of the first diffraction orders (Fig. 5c): *R*_*−*1_ = 0.2% and *R*_+1_ = 0.4%. Moreover, unlike the first approach, this one clearly reveals the asymmetry in the diffraction efficiencies obtained in the experimental measurements. Noted above good qualitative and even quantitative correspondence between the measured diffraction efficiencies and those estimated from the linearized SDHM response indicates that the SDHM characterization provided accurate information on the phase-amplitude profile of the binary grating.

**Figure 7:**
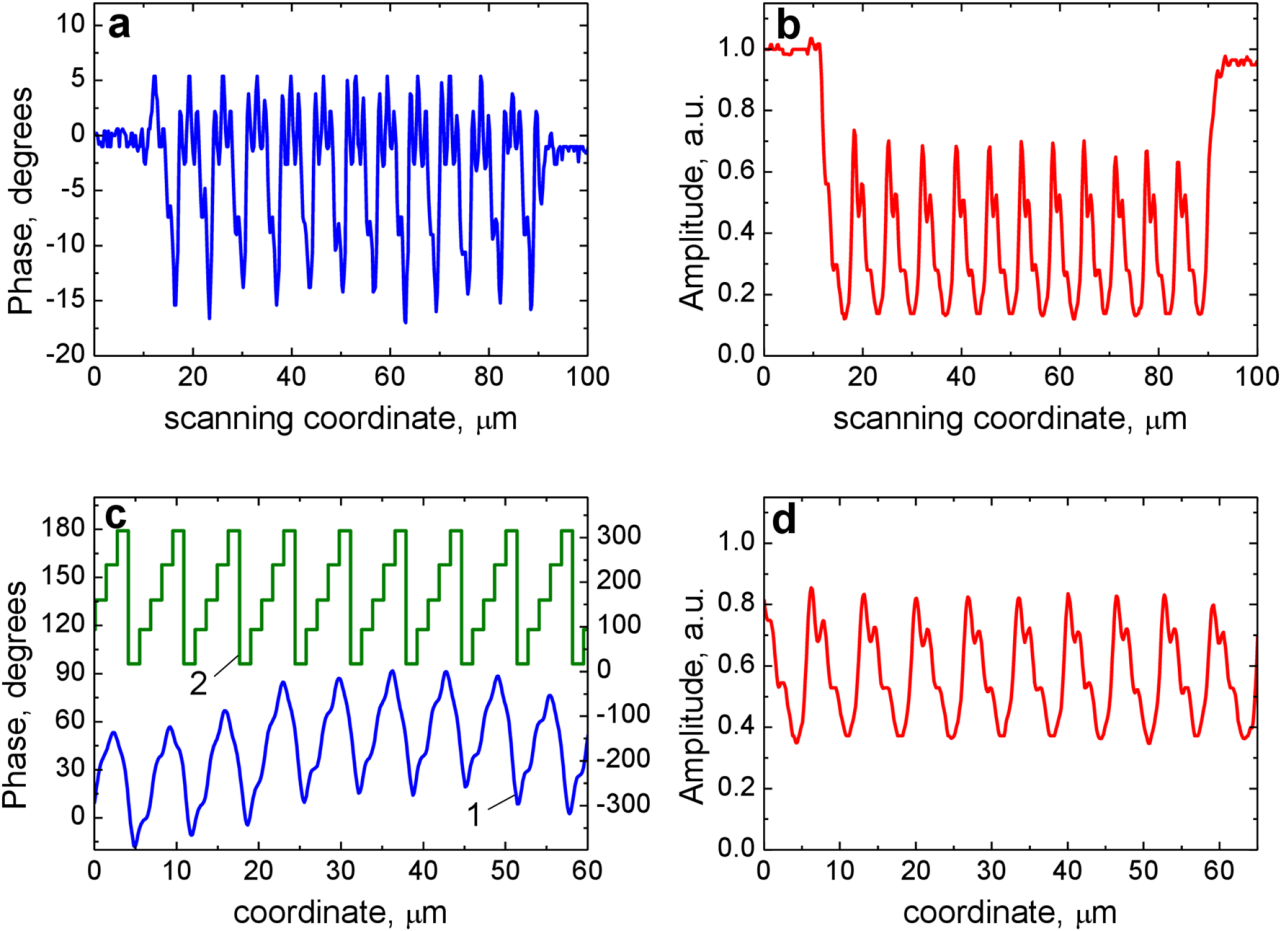
Phase-gradient metasurface object profile retrieval based on the linearization algorithm. (**a**) SDHM phase response to the phase-gradient metasurface. (**b**) SDHM amplitude response to the phase-gradient metasurface. (**c**) Linearized phase response (1, left *Y* axis) and designed phase profile of phase-gradient metasurface (2, right *Y* axis). Five phase levels of metasurface profile are specified in the caption to Fig. 3b. (**d**) Linearized amplitude response.

**Figure 8:**
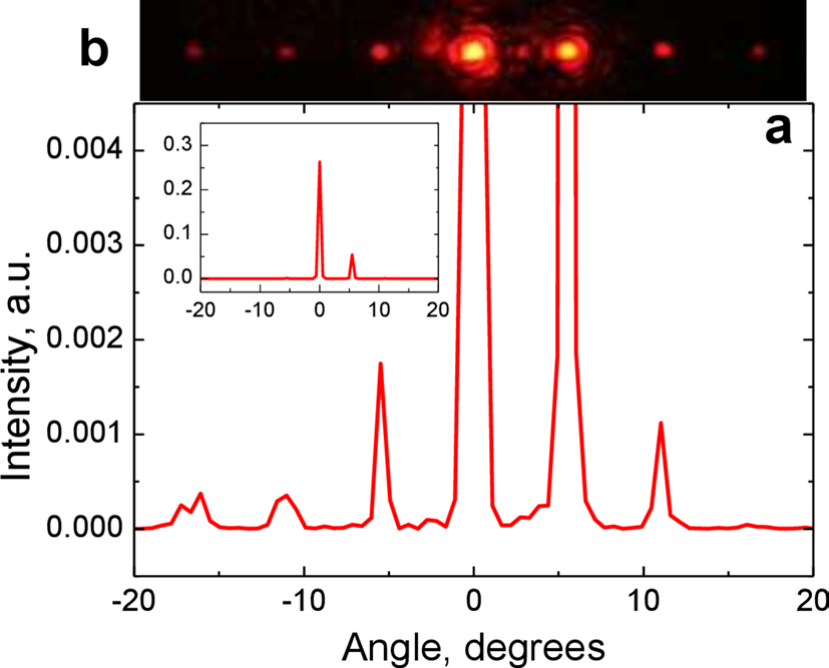
(**a**) Calculated spectrum intensity for the reconstructed phase-gradient metasurface profile. The full-scale intensity is shown in the inset. (**b**) Experimentally registered diffraction pattern of the phase-gradient metasurface.

The second object under investigation was the phase-gradient metasurface (Fig. 3e). The set of individual cells has a length of 1.35 μm, the grating period (i.e. the supercell length) is *P*_2_ = 6.75 μm. The complex response of SDHM to the phase-gradient metasurface is shown in Fig. 7a,b. Estimation of the amplitude-phase profile by Eq. (3), in this case may be incorrect, since the microscope does not completely resolve steps spaced by 3Λ = 1.35 μm. Therefore, for this object we can use only the second method (i.e. linearization). The linear response of the microscope, which should represent the amplitude-phase profile of the grating, is shown in Fig. 7c,d. The calculation of the diffraction efficiency gave the following results: *R*_0_ = 26%, *R*_−1_ = 0.2%, *R*_+1_ = 5.5% (Fig. 8a). The experimental diffraction pattern is shown in Fig. 8b. Experimental measurements of diffraction orders gave the following results: *R*_0_ = 21%, *R*_−1_ = 0.3%, *R*_+1_ = 9%. The main characteristic of such devices is the contrast *γ* as a measure of the efficiency difference in diffraction orders. The contrast of the grating diffraction pattern related to the 1st orders is given by: *γ* = *R*_+1_/*R*_−1_. In our case, *γ* = 30 for direct diffraction measurement and *γ* = 28 for SDHM data. We should note that overall measured diffraction pattern and calculated one (Fig. 8) coincide quite accurately. For example, even the splitting of the − 3rd diffraction order is clearly seen both on experimentally registered (Fig. 8b) and calculated (Fig. 8a) diffraction patterns. At the same time it is worth noting the characteristic "arched" baseline of the linear phase responses (Figs. 6a and 7c). Most likely, it appears due to phase noise and/or grating heterogeneity associated with the imperfection of the manufacturing process and should be investigated further.

## Discussion

The presented study is devoted to experimental verification of efficiency and accuracy of the SDHM characterization for direct inspection of GSP reflective metasurfaces, using as a starting point the analytical considerations and numerical calculations reported previously^10^. Two GSP-based metasurface configurations, representing a binary grating and linear phase gradient, were experimentally characterized with the SDHM operating at the light wavelength of 633 nm. The phase-amplitude reflection profiles of these surface nanostructures were reconstructed from the experimentally obtained SDHM responses and used to calculate the expected diffraction efficiencies that have also been measured in the carefully set up diffraction experiment. Comparing the experimental performances of the fabricated metasurfaces with those expected from the phase-amplitude profiles reconstructed from the SDHM measurements, we found their good qualitative and quantitative correspondence, verifying thereby the efficiency and accuracy of the developed SDHM characterization approach for direct inspection of GSP reflective metasurfaces.

The obtained experimental results open up the possibility of various applications of SDHM in solving the problem of characterization of plasmon metasurfaces. First of all, it should be noted that even the unprocessed initial phase-amplitude response of the SDHM allows one to obtain important information about the degree of homogeneity of the metasurface under study in its various sections, with displacements multiple of the period. For the perfect step structure (Fig. 4c,d) all response details within each period are equal (Fig. 4e,f) and in SDHM response to the experimental structures (Figs. 4a,b and 7a,b) the difference between various period areas is clearly seen. Further processing SDHM response using linearization algorithm makes it possible to evaluate the optical reflection profile of metasurface structures. For example, the periodic sawtooth profile of the reconstructed amplitude-phase profile of the metasurface obtained using the SDHM technique (Fig. 7c) is in excellent agreement with the treatment of the phase-gradient metasurface as a blazed grating^11^. The revealed good agreement between the calculated diffraction patterns in the far-field and the experimentally measured one confirms the good perspectives to use the proposed method for the practical characterization of plasmon diffraction metasurfaces.

Concerning technical limitations in using the SDHM technique for metasurface inspection, the main and most fundamental limitation is related to the resolution limit that any far-field microscopy technique is subjected to. In principle, this limitation can be circumvented, at least to some extent, by increasing the signal-to-noise ratio along with using *apriori* information about the metasurface under inspection and the SDHM response function^10^. The most relevant technical limitation is thereby related to the fundamental limit of signal-to-noise ratio that is difficult to achieve in practice when using moderately powerful lasers. There are also technical limitations related to the accuracy of scanning and data acquisition as well as a general stability of the optical setup employed, which are common to all scanning optical microscopy techniques. In our case, we are far away from fundamental signal-to-noise ratio limits, and the accuracy of measuring the phase-amplitude SDHM response can be increased by at least the order of magnitude with upgrading the technical equipment used. Also, it should be mentioned that our SDHM measurements are limited by using only one wavelength and recording 1D (one-dimensional) phase-amplitude profiles. Both technical limitations can be eliminated by adding additional coherent optical sources to the SDHM optical configuration and extending the scanning capabilities. Even though the SDHM technique due to its far-field nature does not have enough resolution to individually characterize each (subwavelength-sized) unit cell of a metasurface, it can still provide accurate information on the phase-amplitude response of a (periodically repeated) supercell that determines the practically relevant functionality of a metasurface as demonstrated in our work.

Concluding, we would like to emphasize that, as an optical characterization tool, the SDHM technique possesses all natural advantages of the classical (far-field) scanning optical microscopy^15^, which are very useful in various practical optical inspection applications. In this work, we have experimentally demonstrated SDHM technique for characterization of optical gradient metasurfaces operating in reflection. First, we have shown that the difference in optical properties of individual cells forming metasurface caused by technology imperfectness can be detected in the SDHM response. Second, we have shown that by proper analysis of the phase and amplitude data from the SDHM response and subsequent phase-amplitude metasurface profile evaluation, the far-field diffraction pattern for the metasurface can be predicted with good accuracy. Third, we have confirmed the main advantages of SDHM, namely accurate acquisition of the quantitative phase-amplitude data across large field of view accompanied by a simple and robust set-up. The SDHM characterization investigated in this work can thereby be used for direct, efficient and accurate inspection of GSP reflective metasurfaces developed for diverse applications^1^.

## Methods

### Modelling and response processing

For designing metasurface elements, COMSOL Multiphysics software based on finite-element method was used. An individual unit cell was model built in COMSOL with periodic boundary conditions on vertical sides of the cell. The excitation and collection ports were applied from above with perfectly matched layers to minimize reflections. In the excitation port, the incident light was chosen to be a linearly polarized plane wave. The nanobrick corners were 5-nm rounded for better correspondence with fabricated nanobricks. The material data used for gold were taken from Johnson and Christy^18^, and the refractive index of dielectric spacer layer was taken to be 1.45.

Modelling results concerning SDHM response to step-like objects are simulated using the image formation theory for SDHM (see Supplementary, Section 1) and implemented in Matlab. The parameters of simulation are as follows: *λ* = 633 nm, NA = 0.5, *δ* = 0.5 мкм. The linearization procedure consists in solving Eq. S1.2 and is based on the expansion of linear response and differential one according to the Shannon sampling theorem (see Supplementary, Section 2). Here the sampling interval Δ*x* = 0.22 μm and the number of samples 2* N* + 1 = 500, thus the scanning interval of 110 μm covers the object size of 80 μm and the substrate at both sides of the structure.

### Fabrication

The fabrication of the investigated metasurfaces is carried out following the same procedure and using the same equipment as those used for obtaining the previously published results on plasmonic phase-gradient metasurfaces^12^. The GSP-based metasurfaces are fabricated using electron-beam lithography and lift-off technique. On a base substrate of glass 100 nm of gold is deposited by e-beam evaporation and subsequently 40 nm of SiO_2_ is deposited by RF-sputtering in a Cryofox 600 system equipped with a Temescal SuperSource2 e-beam source and a MAK magnetron RF-sputter source. Following a spin coating of 120 nm PMMA 950 K A2, the gold nanobrick structures with an interparticle distance of 450 nm are lithographically defined by e-beam lithography and formed by e-beam evaporation of 50 nm gold followed by a lift-off process.

The nanobricks are arranged in 80 × 80 *μ*m^2^ arrays and they are exposed at 30 kV using a JEOL-640LV electron microscope equipped with an ELPHY Quantum lithography system.

### Response measurement

In the SDHM scheme a He–Ne laser (*λ* = 633 nm) has an output optical power of 30 mW. The diffraction efficiency at the Bragg cell in first order is about 10%. Optical interference signal is registered by a PIN diode. The parameters of the diode output signal are measured by selective voltmeter and phasemeter during scanning the object placed on *XYZ* translation stage. The stage is driven by a stepper motor with a speed reducer shifting the object along *X* axis. The scanning step and the beam separation are both equal to 0.22 μm. Analogue outputs of the voltmeter and phasemeter are converted by ADC and then entered into a computer. The accuracy of the phase signal being measured is 0.2°. For the normalized amplitude signal the accuracy is 0.01.

### Diffraction efficiency measurement

To measure the diffraction orders the optical scheme is designed and built specially for this purpose (see Supplementary, Section 3). The same He–Ne laser is used as a light source. The scheme forms a plane wave incident on the metasurface which should be located in the front focal plane of the microobjective. The diffraction pattern is formed in the far-field at the distance of ~ 2 m from the microobjective. The distance of 7 m to the photomultiplier is needed to achieve an appropriate optical magnification.

## Supplementary information

Supplementary file1 (PDF 325 kb)
