# Supplementary material for: Characterization of gap-plasmon based metasurfaces using scanning differential heterodyne microscopy
Source: Sci Rep. 2020 Aug 11;10:13524. doi: 10.1038/s41598-020-70395-2 (PMC7419529; doi:10.1038/s41598-020-70395-2)
Supplement: Supplementary file 1 — Supplementary file1 (PDF 325 kb) [file 41598_2020_70395_MOESM1_ESM.pdf]

SUPPLEMENTARY INFORMATION

Characterization of gap-plasmon based metasurfaces using scanning differential heterodyne microscopy

Ildar M. Akhmedzhanov, Rucha A. Deshpande, Dmitry V. Baranov & Sergey I. Bozhevolnyi

1. Image formation theory

The microscope response is calculated with the image formation theory of scanning differential heterodyne microscope (SDHM) in the thin phase screen approach.[1] This theory makes it possible to obtain the dependence of the photodetector current on the amplitude-phase reflective characteristics of the object under study and the signal recording conditions. The main peculiarity of the SDHM in this scheme is the registration of the interference signal at the heterodyne frequency with a point photodetector in the Fourier plane. Thus, the selected registration conditions allow optimizing the photocurrent amplitude within coherent image formation conditions. This makes possible detecting both components of harmonic photocurrent, namely the phase and amplitude. According to the image formation theory the photocurrent is given by

$$i(x_s,t) = i_0 \operatorname{Re}\{D(x_s)\exp(2\pi j f_i t)\}, \tag{1.1}$$

where

$$D(x_s) = L(x_s+\delta/2)L^*(x_s-\delta/2), \tag{1.2}$$

L is the normalized electric field related to reflected field of one beam, D is the interference reflected intensity of two beams, δ is the interval between focused beams in the object plane, x_s is the scanning coordinate, f_i is the heterodyne frequency, i_0 is the photocurrent for an unpatterned surface. Usually we call the function L as a linear response and the function D as a differential response. Both functions can be described as a complex function because the harmonic current is completely characterized by its amplitude and phase.

For mentioned registering conditions the SDHM linear response is a analogue of a image or response in a conventional scanning microscope and can be written as[2]

$$L(x_s) = \int_{-\infty}^{\infty} g(x)r(x-x_s)dx, \tag{1.3}$$

where

$$g(x) = \frac{1}{\pi x}\sin\left(\frac{2\pi x}{\lambda}\mathrm{NA}\right) \tag{1.4}$$

is the line spread function, $r(x)$ is the reflection coefficient of an object, NA is the numerical aperture of the microobjective. Here the line spread function operates instead the point spread function due to one-dimensional function $r(x)$. Thus Eqs. 1.1–1.4 allow to simulate SDHM response within the thin phase screen approach.

2. Response linearization

The linearization of the SDHM response $D(x)$ is a procedure to determine the unknown function $L(x)$ provided the known function $D(x)$ using Eq. 1.2. In the procedure the Shannon sampling theorem[3] is used. According to the theorem the functions D and L can be defined through their values at samples x_n as

$$D(x) = \sum_{n=-\infty}^{\infty} D_n S_n(x), \qquad L(x) = \sum_{n=-\infty}^{\infty} L_n S_n(x-\delta/2), \tag{2.1}$$

where

$$S_n(x) = \frac{\sin \pi (x - x_n) / \Delta x}{\pi (x - x_n) / \Delta x}$$

is the sampling function, $D_n = D(x_n)$, $L_n = L(x_n + \delta/2)$, $x_n = n\Delta x$, Δx is the sampling interval. Substituting (2.1) to (1.2) at $x = x_n$ and $\Delta x = \delta$ we get the recurrent equation:

$$D_n = L_n L^*_{n-1} . \quad (2.2)$$

To solve Eq. 2.2 we represent the complex samples D_n and L_n in the exponential form:

$$D_n = |D_n| \exp[i\Phi_n], \quad L_n = |L_n| \exp[i\varphi_n]. \quad (2.3)$$

Taking into account the initial condition: $|L_{-\infty}| = 1$ and $\varphi_{-\infty} = 0$ for normalized response, the solution for a set of the limited number $2N + 1$ of samples can be written as:

$$\varphi_n = \sum_{m=-N}^{n} \Phi_m, \quad n = -N, -N+1, \ldots N \quad (2.4)$$

$$|L_n| = \frac{|D_n||D_{n-2}||D_{n-4}|\cdots}{|D_{n-1}||D_{n-3}|\cdots} . \quad (2.5)$$

In this paper we do use Eq. 2.4 for phase samples and do not use Eq. 2.5 for amplitude samples in the linearization algorithm. It is appeared that the calculation a square root of the amplitude response $|D(x)|$ to determine amplitude linear response $|L(x)|$ is more correct than using Eq. 2.5 if the spot distance δ is much less than the spot size $2w$ (i.e. $\delta << 2w$, see Fig. 2b in the main manuscript). This is due to the presence of a noise component in the experimental response resulting to the sufficient error increase because of numerous divisions in Eq. 2.5.

2. Diffraction measurement

The measurement of the diffraction efficiencies have been performed using optical scheme presented in Fig. 1. The half-wave plate (2) and polarizer (3) is used to control the polarization of He-Ne laser (1) emission at $\lambda = 633$ nm. The output optical power of the laser is 30 mW. The focal planes of the lens (6) and microobjective (7) are coincided to form a plane wave incident on the grating. The iris diaphragm adjusts the beam diameter to match incident spot with grating size. The grating is mounted on a *XYZ* translation stage and located in the focal plane of the microobjective. The diffraction pattern is formed in the far-field zone and the photomultiplier is used to measure the intensity of diffraction spots with high dynamic range (>50 dB).

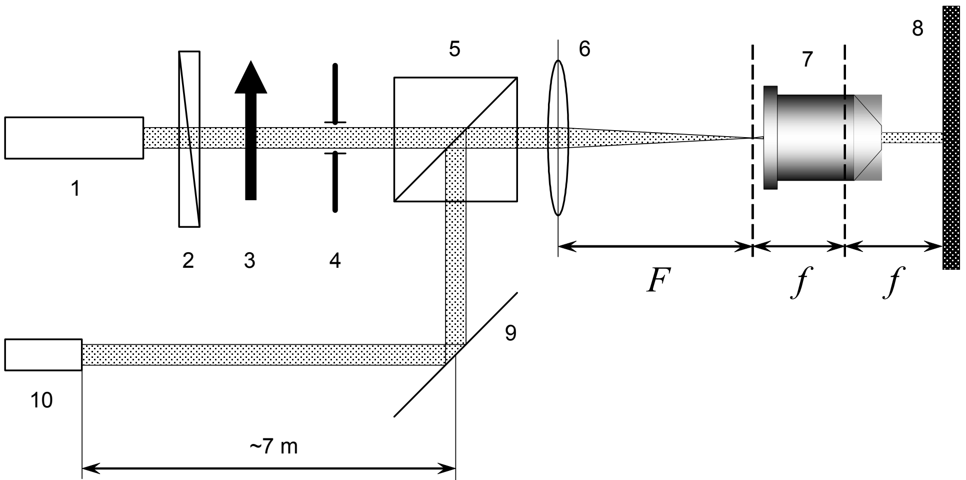

Figure 1. Sketch of the optical scheme to measure diffraction efficiencies. (1) He-Ne laser ($\lambda = 633$ nm); 2 – half-wave plate; 3 – polarizer; 4 – iris diaphragm; 5 – splitter; 6 – lens ($F = 36$ cm); 7 – microobjective $40^{\times}/0.65$ ($f = 4.3$ mm); 8 – grating; 9 – mirror; 10 – photomultiplier.

References

1. Baranov, D. V. & Zolotov, E. M. Superresolution processing of the response in scanning differential heterodyne microscopy in *Advances in information optics and photonics* (eds. Friberg A. T. & Dandliker R.) 229–250 (SPIE Press, 2008).
2. Wilson T. & Sheppard C. J. R. *Theory and practice of scanning optical microscopy* (Academic press, 1984).
3. Papoulis, A. *Systems and Transforms with Applications in Optics* (McGraw-Hill, 1968).
